# The Italian guideline on comprehensive geriatric assessment (CGA) for the older persons

**DOI:** 10.1007/s40520-024-02780-0

**Published:** 2024-05-27

**Authors:** Stefania Maggi, Luigi Ferrucci

**Affiliations:** 1 CNR Aging Branch, Neuroscience Institute, Padua, Italy; 2 Intramural Research Program of the National Institute On Aging, NIH, Baltimore, MD USA

The Guideline on comprehensive geriatric assessment (CGA) for the Older Persons, published in the present issue of *Aging Clinical and Experimental Research*, is perhaps the first attempt to summarize the massive literature on this topic that has been published over the last decade, with a comprehensive approach never attempted before [1]. This highly needed and long-awaited operational document shall guide clinical and public health effort to respond to the new needs of medical and social support that is emerging from a population that is fast aging. The standardization of CGA in older adults across different settings is particularly important in countries like Italy, that have the oldest world population and where broader implementation of CGA also outside traditional geriatric settings has become a health priority that cannot longer be delayed.

Despite decades of CGA experience in clinical and research field [2], the culture on the CGA is still not robustly rooted in different geographical areas, settings and specialties, with substantial heterogeneity of approaches and implementations [3]. The availability of new comprehensive document should allow a proper standardization of diagnostic workflow and define, based on patient’s prognosis through CGA-based tools, personalized care plan, appropriate monitoring of treatment outcomes and, ultimately, a better allocation of resources. We particularly appreciated the fact that the guideline highlighted the role of CGA in reducing the risk of negative outcomes in different areas of care, i.e., hospitalization, institutionalization, delirium, drug prescription inappropriateness, worsening of functional activities and mortality. It is somewhat disappointing that some recommendations are not supported by strong scientific evidence. While we recognize that gathering evidence in this research is difficult, in part because of the great heterogeneity of study design and operational CGA methodology reported in different settings and organizations of the health systems worldwide, the lack of evidence should be a stimulus to conduct more research in this critical area. Indeed, the effects of CGA in some settings as rehabilitative facilities, nursing homes, hospice and palliative-care appeared still poorly explored. This fact does not make the Guideline less valid but, on the contrary, stresses the need of a huge and capillary work for spreading and monitoring the implementation and the efficacy of the CGA over the next years.

A highlight of this work is the great attention paid to the description and role definition of different health figures from different disciplines in the panel of experts, so that the perspectives of the various professionals involved in the management of older patients could be efficiently represented. Moreover, the inclusion of representatives of patients and civil society may enhance understanding of how patients perceive different procedures and aspects of organization and how patient perspective can be better introduced in the definition of personalized care plans and treatment objectives.

There is no doubts that older patients will rise to be the overall majority of patients accessing a health care systems that is unprepared to respond to their special demands. Guideline, even with still several open questions, must rise a sense of urgency in all the stakeholders, and increase awareness of the consequences that may follow by not implementing such multidimensional approach both in the Italian and global population of older adults. Now, there is a need for a step forward, with the establishment of a task force at the National Institute of Health, that should monitor the use of this Guideline and gather information on potential barriers or difficulties in the implementation of CGA approach to the older people and gain further insight on the economic impact to the healthcare systems. We need to move from the science of CGA to its wide implementation so we can adapt the principles of CGA to local realities with the goal of providing better care across different contexts.
